# Iron content of ferritin modulates its uptake by intestinal epithelium: implications for co-transport of prions

**DOI:** 10.1186/1756-6606-3-14

**Published:** 2010-04-29

**Authors:** Solomon RB Sunkesula, Xiu Luo, Dola Das, Ajay Singh, Neena Singh

**Affiliations:** 1 Department of Pathology, Wolstein Research Building, Case Western Reserve University, 2103 Cornell Road, Cleveland, OH- 44106, USA

## Abstract

The spread of Chronic Wasting Disease (CWD) in the deer and elk population has caused serious public health concerns due to its potential to infect farm animals and humans. Like other prion disorders such a sporadic Creutzfeldt-Jakob-disease of humans and Mad Cow Disease of cattle, CWD is caused by PrP-scrapie (PrP^Sc^), a β-sheet rich isoform of a normal cell surface glycoprotein, the prion protein (PrP^C^). Since PrP^Sc ^is sufficient to cause infection and neurotoxicity if ingested by a susceptible host, it is important to understand the mechanism by which it crosses the stringent epithelial cell barrier of the small intestine. Possible mechanisms include co-transport with ferritin in ingested food and uptake by dendritic cells. Since ferritin is ubiquitously expressed and shares considerable homology among species, co-transport of PrP^Sc ^with ferritin can result in cross-species spread with deleterious consequences. We have used a combination of *in vitro *and *in vivo *models of intestinal epithelial cell barrier to understand the role of ferritin in mediating PrP^Sc ^uptake and transport. In this report, we demonstrate that PrP^Sc ^and ferritin from CWD affected deer and elk brains and scrapie from sheep resist degradation by digestive enzymes, and are transcytosed across a tight monolayer of human epithelial cells with significant efficiency. Likewise, ferritin from hamster brains is taken up by mouse intestinal epithelial cells *in vivo*, indicating that uptake of ferritin is not limited by species differences as described for prions. More importantly, the iron content of ferritin determines its efficiency of uptake and transport by Caco-2 cells and mouse models, providing insight into the mechanism(s) of ferritin and PrP^Sc ^uptake by intestinal epithelial cells.

## Background

Prion disorders are a group of neurodegenerative conditions of humans and animals that are known to be transmitted through ingestion of prion contaminated material. This mode of transmission was described historically as 'Kuru', a neurodegenerative condition of humans acquired by ingesting prion disease affected human brain tissue [1,2]. Later, the disease was transmitted to humans from diseased cows, referred to as variant Creutzfeldt-Jakob disease (vCJD) [3-5]. Despite convincing evidence of its transmission through contaminated food, the mechanism by which PrP-scrapie (PrP^Sc^), the principal pathogenic and infectious agent responsible for all prion disorders crosses the stringent epithelial cell barrier has remained enigmatic. Transport through dendritic cells, M cells, and co-transport in association with ferritin has been reported, but a complete understanding of either pathway is lacking [6-8].

PrP^Sc ^is a β-sheet rich isoform of a normal cell surface glycoprotein, the prion protein (PrP^C^) that acquires certain biochemical characteristics such as insolubility in non-ionic detergents, tendency to aggregate, limited resistance to digestion by proteinase-K, and the ability to replicate [9]. Most infectious prion disorders are acquired when PrP^Sc ^from an exogenous source gains access to the central nervous system and induces the conversion of host PrP^C ^to its own conformation. A certain amount of homology between the incoming PrP^Sc ^and host PrP^C ^is required for efficient conversion, explaining the protective influence of species barrier such as between rodents and humans [10]. However, the possible transmission of sheep scrapie to cattle and onward transmission to humans indicates that non-homologous PrP^Sc ^can be carried by certain hosts, and in some cases, convert host PrP^C ^to a novel PrP^Sc ^conformation, albeit very slowly [11-13]. In view of these facts, it is important to evaluate whether PrP^Sc ^from deer and elk population infected with CWD can cross the species barrier and create a carrier state in cattle sharing the same grazing ground, or through contaminated food products in humans [14-17].

Since the most likely source of natural infection with CWD and other prion disorders is through ingestion of PrP^Sc ^contaminated food, it is important to understand the mechanism by which PrP^Sc^, a protein of 27-37 kDa survives the harsh digestive environment and crosses the stringent epithelial cell barrier while retaining its infectious nature. The resilience of PrP^Sc ^to digestive enzymes is shared with ferritin, an iron storage protein that is a common constituent of all foods. In a previous report we demonstrated that PrP^Sc ^forms a relatively stable complex with ferritin in prion disease affected brain homogenates, and the complex is transcytosed together across a monolayer of Caco-2 cells, an *in vitro *model of human epithelial cell barrier [7,18,19]. Since ferritin shares significant homology across species, PrP^Sc ^from distant species is likely to ride 'piggy back' on ferritin to cross the epithelial cell layer, raising the possibility that infectious PrP^Sc ^from distant species such as deer and elk could cross the intestinal epithelial barrier of cattle or humans, and create a carrier state [20,21].

To evaluate this possibility, we have checked the transport of ferritin from different species across a tight monolayer of Caco-2 cells, and confirmed our observations *in vivo *in mouse models. We report that brain ferritin from three different cases of CWD and sheep scrapie resists degradation by digestive enzymes (DE) and is associated with PrP^Sc^. Both ferritin and PrP^Sc ^from diseased CWD and sheep brains are transported across a monolayer of Caco-2 cells, and the iron content of ferritin determines its efficiency of uptake and transport by Caco-2 cells *in vitro*, and by mouse intestinal epithelial cells *in vivo*. These observations have significant implications for inter-species spread of CWD and other animal prion disorders.

## Methods

### Chemicals and antibodies

Anti-PrP antibody 3F4 was obtained from Signet Laboratories (Dedham, MA, USA), anti-ferritin antibody from Sigma (St. Louis, MO, USA), anti-zonula occludens-1 (ZO-1) from Zymed (San Francisco, CA, USA), and horseradish peroxidase (HRP)-conjugated secondary antibodies were obtained from GE Healthcare (Little Chalfont, Buckinghamshire, UK). Goat anti-Rabbit FITC (fluorescein isothiocyanate) and TRITC (tetramethylrhodamine B isothiocyanate) labeled secondary antibodies were from Southern Biotechnology Associates (Birmingham, AL, USA). Sulfo-NHS-biotin and streptavidin-Texas Red were purchased from Pierce (Rockford, IL, USA). All cell culture supplies were obtained from Invitrogen (Carlsbad, CA, USA). Nuclear stain Hoechst 33342 was obtained from Molecular Probes (Eugene, OR, USA). Radiolabeled ^59^FeCl_3 _was purchased from Perkin Elmer (Boston, MA, USA). All other reagents including desferroxamine (DFO) were procured from Sigma.

### Preparation of brain homogenates, treatment with DE and proteinase K and immunoprecipitation

A 10% homogenate of normal, CWD, sheep scrapie, or sporadic CJD (sCJD) brain tissue was prepared in phosphate buffered saline (PBS, pH 7.4) by sonication on ice. *In vitro *digestion with enzymes was carried out as described in a previous report [7]. Briefly, 0.5 ml of homogenate was incubated with 200 U of salivary amylase for 15 min at 37°C. The pH of the mixture was adjusted to 2.0 with 5 M HCl followed by addition of 50 μl of pepsin (4095 U) and incubation for 1 h at 37°C. Subsequently, the pH was raised to 6.0 with 1 M sodium bicarbonate and 0.2 ml of pancreatin-bile extract was added (1.85 mg of pancreatin and 11 mg of bile extract/ml of 0.1 M NaHCO_3_). After raising the pH to 7.4 with 6N NaOH, 8.4 μl each of 2 M NaCl and KCl solutions were added and the mixture was rocked for additional 2 h at 37°C. Digestive enzymes were inactivated by the addition of PMSF and protease inhibitors and samples were stored at -70°C till further use.

For PK treatment, the homogenate was mixed with equal volume of 2× lysis buffer (20 mM Tris-HCl; pH 7.4, 100 mM NaCl, 10 mM EDTA, 1% NP-40, and 0.5% sodium deoxycholate) and incubated with 50 μg/ml of proteinase K (PK) for 1 h at 37°C. The reaction was stopped by the addition of PMSF and protease inhibitor cocktail. For co-immunoprecipitation studies, untreated or PK- or DE-treated normal homogenate (NH) and sCJDH samples were immunoprecipitated with anti-ferritin antibody as described previously, and eluted proteins were subjected to Western blotting and probed with 8H4, a monoclonal antibody specific to PrP [7].

### Transport of PrP^Sc ^across Caco-2 monolayers

Caco-2 cells were cultured as described in an earlier report [7]. In short, cells were resuspended in DMEM at a density of 2 × 10^8 ^cells/ml and added to the apical chamber (AP) chamber of poly-carbonate filters (Trans-well; 12 or 24 mm diameter; 3 μm pore size; Costar, Cambridge, MA) coated with collagen. The filters were placed in a 12- or 6-well culture dish containing 0.6 or 1.2 ml of DMEM respectively. The medium was replaced every day until the development of confluent monolayers with tight junctions (10-14 days). The integrity of tight junctions was monitored by measuring trans-epithelial electrical resistance (TEER) across the monolayer with a millicell-ERS instrument (Millipore, Bedford, MA). Monolayers exhibiting a TEER of > 400 Ώ/cm^2 ^were used for transport studies.

To evaluate ferritin and PrP^Sc ^transport, Caco-2 monolayers were washed with serum-free medium, and 20 μl of sample diluted in 1 ml of serum-free medium was added to the AP chamber. The inserts were placed in a 6-well dish containing 1.2 ml of serum-free medium. After an overnight incubation at 37°C, medium from AP and basolateral (BL) chambers were collected, clarified for cell debris, and proteins were precipitated with cold methanol. Precipitated protein were boiled in sample buffer, immunoblotted, and detected with specific antibodies as described below.

### Radiolabeling of cellular ferritin with ^59^FeCl_3 _and transport

Radiolabeling of mouse neuroblastoma N2a cells was performed essentially as described previously [22]. Briefly, N2a cells cultured to 80% confluence were incubated in serum free medium for 1 h followed by incubation with ^59^FeCl_3_-citrate complex (1 mM sodium citrate and 20-25 μCi of ^59^FeCl_3 _in serum free Opti-MEM) for 4 h at 37°C. Following the incubation, cells were washed with ice cold PBS and lysed in PBS or native lysis buffer (0.14 M NaCl, 0.1 M HEPES, pH 7.4, 1.5% Triton X-100 and 1 mM PMSF). The amount of radioactivity incorporated was determined in cell lysates by γ-counting.

All animal procedures were performed as per guidelines established by the Animal Resource Center, Case Western Reserve University, and were based on protocols approved by the IACUC committee. Mice were fed with free ^59^FeCl_3 _or ^59^FeCl_3_-labeled N2a homogenates as described in a previous report [23]. In short, Four months old FVB/NJ mice (Jackson Laboratory) were fasted overnight with water *ad libitum *and equal counts (300,000) of either free ^59^FeCl_3 _mixed with N2a cell homogenate or ^59^FeCl_3 _labeled N2a homogenates in (PBS) were administered orally using an olive-tipped gavage needle. After a chase of 4 h, mice were euthanized and blood was collected in heparinized vials. Brain, liver, spleen, and upper gastro-intestinal tract were harvested, washed with PBS, and snap frozen on dry ice. The organs were weighed, and incorporated radioactivity was counted in a γ-counter. Proximal region (1-2 cm) of duodenum was homogenized in native lysis buffer and analyzed for ^59^Fe labeled ferritin after resolving on native gradient gel followed by autoradiography. Native gradient gel electrophoresis of duodenum homogenates or N2a cell lysates for ^59^Fe-ferritin analysis was done as described by Vyoral et al. [24] with modification as in previous reports [22,23].

### Uptake of biotinylated proteins by mouse intestinal epithelium

Proteins in brain homogenates were biotinylated by adding 1 mg/ml EZ-Link Sulfo-NHS-Biotin followed by an overnight incubation on a rocking platform at 4°C. Biotinylated proteins were concentrated using methanol and suspended in 1 ml of 10% normal brain homogenate. Porcine bile extract was added to the suspended homogenate (11 mg of bile extract/ml of 0.1 M NaHCO3, pH 7.4), and contents were sonicated on ice. This comprised the control sample (-DFO). To deplete the sample of iron, brain homogenate was supplemented with iron chelator DFO (30 μM) and rocked for 1 h at 4°C. Following extensive dialysis against PBS at 4°C to remove DFO-iron complexes, the homogenate was biotinylated and prepared as above, and labeled as +DFO sample. FVB/NJ mice starved overnight were fed 0.2 ml of -DFO or +DFO homogenates by gastric gavage as above. After a chase of 2 h, the mice were sacrificed, and segments of intestine were processed for cryosectioning in Tissue tek OCT compound (Sakura Finetek USA Inc; Torrance, California) and snap frozen in isopentane cooled in Liquid nitrogen. Sections were cut at 5-10 μm thickness and immunostained.

### Immunofluorescence staining

Sections of mouse intestine were permeabilized with cold acetone at -20°C for 1 min and fixed in methanol for 20 min at -20°C. After rinsing with PBS, sections were incubated for 30 min in blocking buffer (PBS containing 3% non immune goat serum) at room temperature. Subsequently, sections were incubated with Streptavidin-Texas red diluted 1:40 in blocking buffer for 40 min at 37°C in a humidified chamber followed by three quick rinses and incubation with polyclonal anti-ferritin (1:10) antibody followed by goat anti rabbit FITC-conjugated secondary antibodies (1:20) for 40 min each. The sections were rinsed in PBS and incubated with monoclonal anti-ZO-1 (1:10) followed by anti-mouse TRITC conjugated secondary antibody (1:20). Stained sections were incubated with Hoechst 33342 (1 μg/ml) for 5 min to detect nuclei and mounted in Gel-mount (Biomeda Corp., Foster City, CA). Sections were then imaged by a fluorescent microscope. For immunostaining M17 cells, sub-confluent cultures were exposed to 5 μl of control or iron depleted samples, incubated for the indicated time, and processed for immunostaining as described in previous reports [25].

### Detection of Iron

Prussian blue reaction was performed to detect iron in brain homogenates as described by Smith et al. [26]. In brief, homogenates spotted on a PVDF membrane were immersed in a mixture of acidified potassium ferro- and ferri-cyanide solution (7%) for 20 min followed by washing with deionized water. Blue color indicated reactive iron.

### SDS-PAGE and Western blotting

Proteins were processed for SDS-PAGE and Western blotting as described previously [27,28]. Proteins transferred to PVDF membranes were probed with anti-PrP 8H4 (1:3000) or anti-ferritin antibody (1:1000) followed by horseradish peroxidase conjugated secondary antibody (1:6000). Immunoreactive bands were visualized by ECL detection system (Amersham Biosciences Inc.).

## Results

### CWD and sheep PrP^Sc ^resist *in vitro *digestion and are associated with ferritin

**Figure 1:**
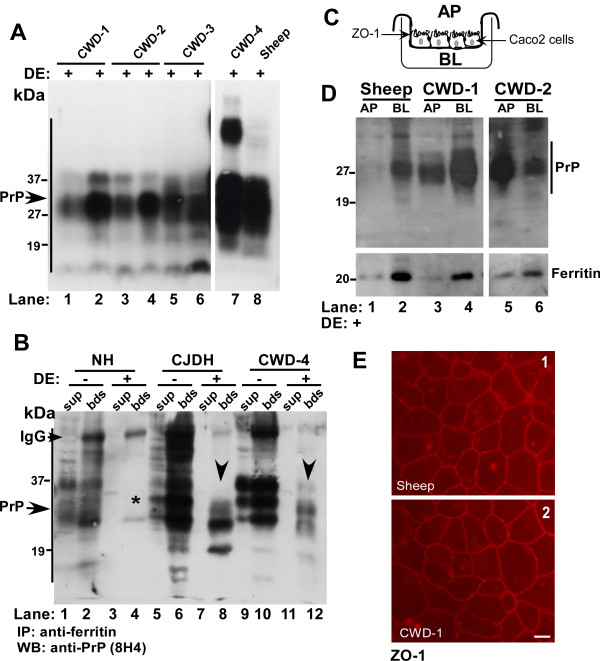
**DE resistant CWD and sheep scrapie are transported across Caco2 monolayers**. **(A) **CWD and sheep scrapie brain homogenates were treated with DE and subjected to immunoblotting. Probing for PrP reveals DE-resistant PrP forms in all four cases of CWD and sheep scrapie (lanes 1-8). (Lanes 1, 3, and 5 were loaded with 5 μl of each sample, lanes 2, 4, and 6 with 10 μl of the same sample, and lanes 7 and 8 with 15 μl of sample to confirm efficient cleavage of PrP^Sc^). **(B) **Control and DE treated NH, CJDH, and CDW-4 samples were immunoprecipitated with anti-ferritin antibody and antibody-protein complexes eluted from beads and unbound proteins in the supernatant were subjected to Western blotting. Probing for PrP shows significant co-immunoprecipitation of PrP with ferritin from DE+ CJDH and CWD-4 samples (lanes 8 and 12). No PrP is detected in DE+ NH samples as expected (lanes 3 and 4, *) and in the supernatant of DE+ CJDH and CWD-4 samples (lanes 7 and 11). Significant amounts of normal PrP and full-length PrP^Sc ^co-immunoprecipitate with ferritin from DE- NH, CJDH, and CWD-4 samples (lanes 2, 6 and 10). **(C) **Model of a trans-well demonstrating the separation of AP and BL chambers by a monolayer of Caco-2 cells. **(D**) DE treated brain homogenates from sheep, CWD-1, and CWD-2 were re-suspended in PBS and added to the AP chamber of a tight monolayer of Caco-2 cells. After an overnight incubation, proteins from AP and BL chambers were precipitated and subjected to Western blotting. Probing for PrP shows significant transport of DE resistant PrP^Sc ^from sheep, CWD-1 and CWD-2 samples to the BL chamber (lanes 1-6). Re-probing for ferritin shows similar transport of ferritin from AP to the BL chamber (lanes 1-6). **(E) **Immunostaining of filter inserts for the tight junction protein ZO-1 shows an intact monolayer of Caco-2 cells with tight junctions (panels 1 and 2). Bar: 10 μm.

Ingested food is subjected to a series of enzymatic reactions beginning with salivary amylase, stomach pepsin, pancreatic enzymes, and bile before absorption by intestinal epithelium. Most proteins are degraded into smaller peptides by this process to facilitate absorption. To evaluate whether PrP^Sc ^from CWD affected deer and elk brains and sheep scrapie resist degradation by digestive enzymes (DE), brain homogenates from four cases of CWD and one case of sheep scrapie were processed for *in vitro *digestion as described [7,29]. Digestion was stopped by addition of protease inhibitors followed by boiling in sample buffer, and 5 and 10 μl aliquots of first three CWD cases and 15 μl aliquots of CWD-4 and sheep samples were fractionated by SDS-PAGE and subjected to Western blotting. Probing with anti-PrP monoclonal antibody 8H4 shows cleaved PrP forms that migrate between 19 and 27 kDa, typical of PK-resistant glycosylated and unglycosylated PrP^Sc ^that accumulates in diseased brains (Figure 1A, lanes 1-8). Although the total amount of PrP^Sc ^is higher in CWD-4 and sheep samples as expected, there is no measurable effect on cleavage efficiency by DE, indicating that majority of PrP^Sc ^is cleaved by this procedure (Figure 1A, lanes 7 and 8). These results suggest that a significant amount of infectious PrP^Sc ^from CWD and sheep brains escapes digestion, and is available for uptake by intestinal epithelial cells.

To evaluate whether PrP^Sc ^from CWD affected brains forms a complex with ferritin as reported for sCJD [7], control and DE treated homogenates from normal (NH), sCJD (CJDH), and CWD-4 sample were immunoprecipitated with anti-ferritin antibody and checked for co-immunoprecipitation of PrP by Western blotting (Figure 1B). Following an overnight incubation with anti-ferritin antibody, protein-antibody complexes were captured with protein-A beads and washed using stringent conditions. Antibody-protein complexes eluted from beads (bds) and unbound proteins in the supernatant (sup) were fractionated by SDS-PAGE, subjected to Western blotting, and probed for PrP with 8H4. A significant amount of PrP from control (DE-) and faster migrating PrP^Sc ^from DE treated (DE+) CJDH and CWD-4 samples co-immunoprecipitates with ferritin (Figure 1B, lanes 2, 6, 8, 10, and 12). As expected, no PrP is detected in DE+ NH samples (Figure 1B, lanes 3 and 4, *), and in the supernatant of DE+ CJDH and CWD-4 samples (Figure 1B, lanes 7 and 11). Full-length PrP migrating between 27-37 kDa is detected in the supernatant and bead fractions of untreated NH, CJDH, and CWD-4 samples, indicating co-immunoprecipitation of normal (PrP^C^) and uncleaved PrP^Sc ^with ferritin (Figure 1B, lanes 1-2, 5-6, and 9-10). No PrP forms were detected in any sample in the absence of anti-ferritin antibody (data not shown). Thus, as demonstrated for sCJD [7], PrP^Sc ^and ferritin form a complex in CWD brain homogenates.

### DE treated CWD and sheep scrapie are transported across Caco-2 monolayers

Although ferritin iron from distant species is taken up efficiently by human intestinal epithelium [30,31], receptors specific for the uptake of ferritin have not been described. Nevertheless, PrP^Sc^-ferritin complex from sCJD brain homogenates is transported across a monolayer of Caco-2 cells [7], suggesting that a similar phenomenon may occur for sheep scrapie and CWD prions. To evaluate this possibility, equal aliquots of DE treated samples from scrapie sheep and two cases of CWD were added to the apical (AP) chamber of a tight monolayer of Caco-2 cells cultured on filter inserts (Figure 1C). Following an overnight incubation at 37°C, culture medium from the AP and basolateral (BL) chambers was harvested, and precipitated proteins were fractionated by SDS-PAGE and transferred to a PVDF membrane. Probing for PrP reveals significant transport of DE-resistant PrP^Sc ^from sheep, CWD-1, and CWD-2 homogenates from the AP to the BL chamber (Figure 1D, lanes 1-6). Reprobing of the membrane for ferritin shows similar transport of ferritin to the BL chamber in all three samples (Figure 1D, lanes 1-6). To rule out possible leakage of proteins from AP to the BL chamber, resistance across filters supporting Caco-2 monolayers was checked before and after completion of the experiment, and the monolayers were stained for ZO-1, a tight junction protein, to confirm the integrity of tight junctions throughout the experimental procedure (Figure 1E, panels 1 and 2).

Together, these results demonstrate that a significant amount of PrP^Sc ^from scrapie infected sheep and CWD affected deer escapes digestion and is associated with ferritin. More importantly, ferritin and PrP^Sc ^from both species are transported across a monolayer of human Caco-2 cells, indicating that the species barrier against animal prions or ferritin is not stringent enough to block transport across these cells.

### Iron content of ferritin determines efficiency of transport across Caco-2 cells

Ferritin is a major iron storage protein ubiquitously present in food products prepared from plant and animal sources, raising the possibility that it could serve as a significant source of dietary iron. Recent reports describe uptake of ferritin through specific receptors on oligodendrocytes, circulating reticulocytes, activated T and B-cells, HeLa cells, and K562 cells [32,33], implicating ferritin as an iron delivery protein. Although the presence of similar receptors on Caco-2 cells is not known, we demonstrated co-transport of PrP^Sc ^and ferritin from sCJD brain homogenates across Caco-2 cells, though the underlying mechanism and whether ferritin or PrP^Sc ^drives transport of the complex was not assessed.

**Figure 2:**
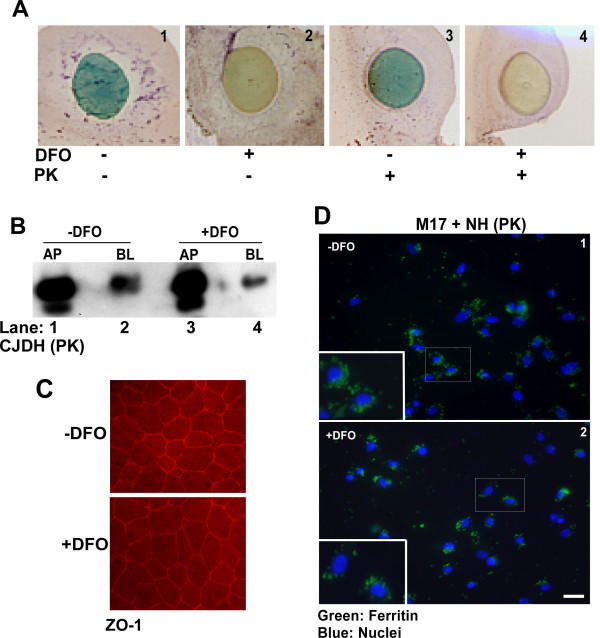
**Iron rich ferritin is internalized by Caco2 and human neuroblastoma cells**. **(A) **Reaction of DFO and PK treated brain homogenate for iron shows significant depletion in the DFO+ sample (panels 1-4). **(B) **Caco-2 monolayers were exposed to PK treated DFO- and DFO+ CJDH in the AP chamber. Following an overnight incubation, precipitated proteins from the AP and BL medium were subjected to Western blotting. Probing for ferritin shows significantly more transport of ferritin to the BL chamber of -DFO sample relative to the +DFO sample (lanes 2 and 4). **(C) **Immunostaining of filter inserts for ZO-1 shows intact monolayer of Caco-2 cells with tight junctions (panels 1 and 2). Bar: 10 μm. **(D) **Human neuroblastoma cells (M17) were exposed to 5 μl of -DFO and +DFO NH sample re-suspended in PBS. Immunostaining of fixed, permeabilized cells with anti-ferritin antibody followed by FITC-conjugated secondary antibody shows prominent reaction for ferritin in intracellular structures consistent with endosomes (panels 1 and 2). Reaction for ferritin is stronger in cells exposed to -DFO sample compared to +DFO exposed cells (panels 1 and 2). Bar: 10 μm. Inset shows enlarged image of the boxed area.

To understand the role of ferritin in this process, transport of untreated control and iron depleted ferritin across a monolayer of Caco-2 cells was checked. To deplete brain ferritin of iron, sCJD brain homogenates prepared in PBS were treated with iron chelator Desferrioxamine (DFO) for one hour, followed by extensive dialysis of untreated (-DFO) and DFO treated (+DFO) samples against PBS. To check the extent of iron depletion, a small aliquot of dialyzed samples was spotted on a PVDF membrane and reacted for iron [26,34,35]. DFO treated sample shows a significant reduction in total iron compared to untreated sample, confirming the chelating effect of DFO (Figure 2A, panels 1 and 2). Subsequently, both samples were treated with proteinase-K (PK) to enrich for ferritin [7]. Most proteins besides ferritin are degraded by this treatment, including disease associated PrP^Sc ^that becomes sensitive to PK following iron depletion [34]. PK-treated samples were checked again for iron content, and no further change was observed by this treatment (Figure 2A, panels 3 and 4). Residual proteins were precipitated with methanol and resuspended in PBS before adding equal amounts of -DFO and +DFO samples to the AP chamber of Caco-2 cell monolayers. Following an overnight incubation, medium from both chambers was collected, and methanol precipitated proteins were resolved by SDS-PAGE followed by Western blotting. Reaction with anti-ferritin antibody shows significantly more transport of ferritin from -DFO sample to the BL chamber compared to the +DFO sample (Figure 2B, lanes 1-4). However, the overall transport of ferritin from AP to the BL chamber is ~5%, suggesting that majority of added ferritin is not transcytosed by these cells. The integrity of Caco-2 monolayers was confirmed as in Figure 1 above before and after completion of the experiment by checking resistance across the monolayer and immunostaining for ZO-1 (Figure 2C, panels 1 and 2).

### Brain ferritin is internalized by neuroblastoma cells

To evaluate whether neuroblastoma cells show similar selectivity for iron rich ferritin uptake as Caco-2 cells, normal human brain homogenate was treated with DFO and PK as above. Subsequently, equal amounts of methanol precipitated proteins from -DFO and +DFO samples resuspended in PBS were added to M17 cells cultured on glass coverslips. After an incubation of 30 minutes at 37°C, cells were washed with PBS, fixed in paraformaldehyde, permeabilized, and immunostained with anti-ferritin antibody followed by FITC-conjugated secondary antibody. A representative image shows significantly more uptake of -DFO treated ferritin by M17 cells relative to +DFO treated sample (Figure 2D, panels 1 and 2).

Taken together, these results suggest that efficiency of ferritin uptake by Caco-2 and neuroblastoma cells is determined in part by its iron content. Subsequent experiments were focused on confirming these results *in vivo *in mouse models.

### Ferritin iron is taken up by mouse intestine

To evaluate whether ferritin is taken up by mouse intestinal epithelial cells, advantage was taken of the fact that ferritin from mouse neuroblastoma cells (N2a) migrates slower than mouse duodenal epithelial cell ferritin when fractionated on a native gradient gel, allowing differentiation between exogenously introduced N2a cell ferritin and endogenous duodenal ferritin.

**Figure 3:**
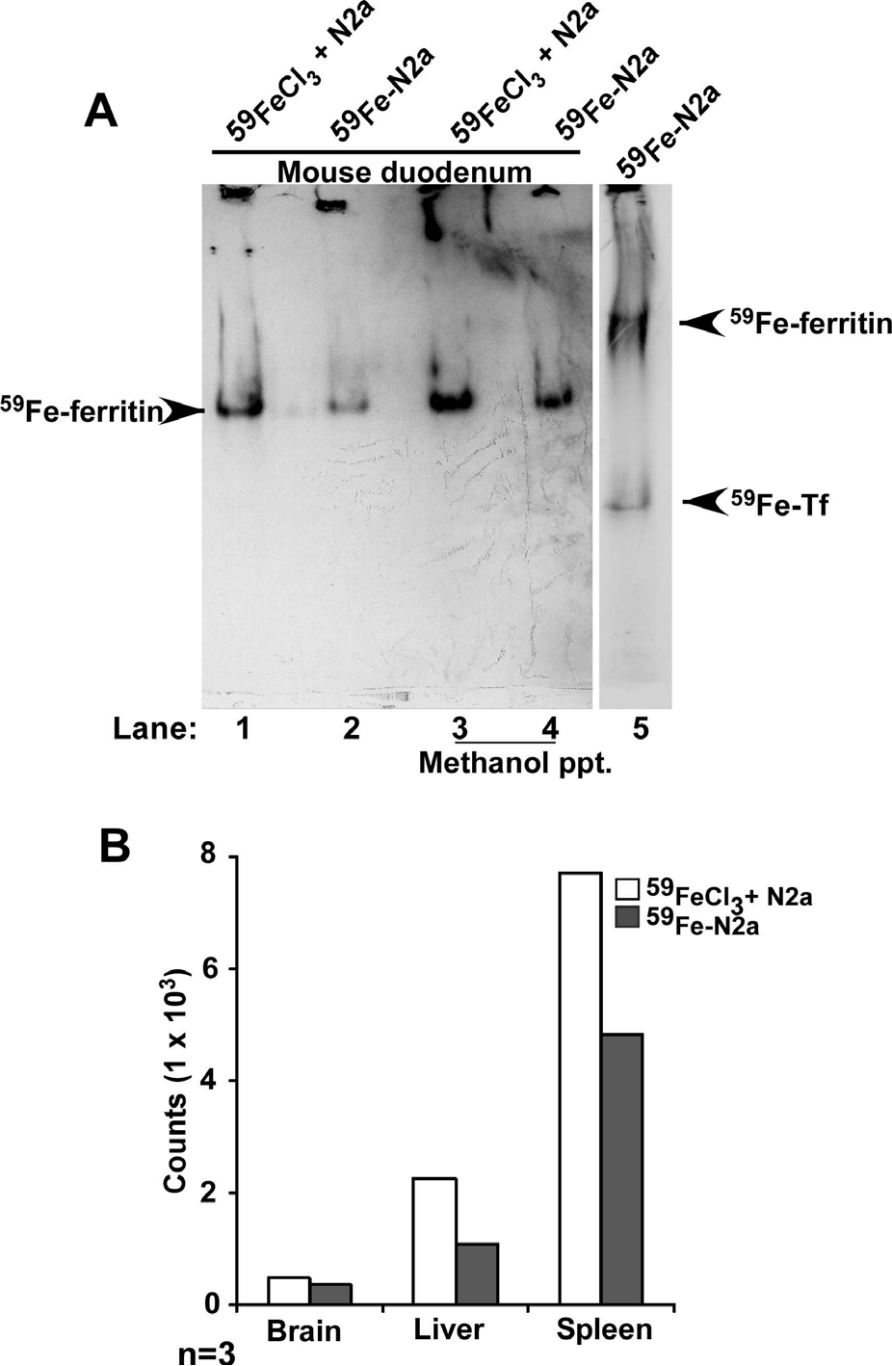
**Ferritin iron is taken up and transported by duodenal epithelium**. **(A) **Wild type mice were fed equal counts of ^59^FeCl_3 _mixed with unlabeled N2a cells (lanes 1 and 3) or ^59^Fe-labeled N2a cells (lanes 2 and 4) and chased for 4 hours. Homogenates of duodenum (lanes 1 and 2) or methanol precipitated protein from duodenum samples (lanes 3 and 4) were fractionated on a native gel and subjected to autoradiography (lanes 1-4). Lysates from ^59^Fe-labeled N2a cells were fractionated in parallel (lane 5). Significantly more ^59^Fe-ferritin is detected in mice fed with ^59^FeCl_3 _relative to ^59^Fe-N2a homogenates (lanes 1 and 3 vs. 2 and 4). The ^59^Fe-ferritin signal is from duodenal ferritin, not N2a ferritin (compare lanes 1-4 with lane 5). ^59^Fe-labeled transferrin (Tf) is also detected in the N2a sample (lane 5) [22,23]**(B) **Quantification of ^59^Fe incorporated by brain, liver and spleen of these mice shows relatively less uptake by mice fed with ^59^Fe-labeled N2a homogenates relative to ^59^FeCl_3_. The data are representative of 3 independent experiments.

Thus, N2a cells were radiolabeled with ^59^FeCl_3 _and the cell pellet was homogenized in PBS. The most abundant protein labeled by this procedure is cellular ferritin. Control sample was prepared by adding same number of ^59^Fe counts (^59^FeCl_3_) to unlabeled cell homogenate prepared under similar conditions. Equal ^59^Fe counts from both samples were introduced by gastric gavage to wild-type mice, and following a chase of 4hours, first 10 cm of the duodenum were harvested. Proximal 2 cm of the duodenum were homogenized in lysis buffer, and a half of each sample was precipitated with methanol to isolate total proteins. The duodenum samples and an aliquot of ^59^Fe labeled cell homogenate were fractionated on a native gel and autoradiographed. Mice fed with ^59^FeCl_3 _mixed with unlabeled homogenate show significantly more ^59^Fe in duodenal epithelial ferritin than the ones fed ^59^Fe-labeled cell homogenate, demonstrating significantly lower efficiency of iron uptake from ferritin relative to ^59^FeCl_3 _(Figure 3A, lanes 1 and 3 vs. 2 and 4). The radioactive bands in lanes 1-4 represent duodenal ferritin, not cellular ferritin that shows a different migration on these gels (Figure 3A, lane 5). ^59^Fe incorporation in major organs of mice fed ^59^FeCl_3 _or ^59^Fe-N2a ferritin show a similar deficiency of iron incorporation by the brain, liver, and spleen of mice fed ^59^Fe-N2a ferritin, consistent with uptake from the intestine (Fig 3B). Since duodenal samples do not show the slower migrating N2a ferritin band, either iron from N2a ferritin is released in the intestinal lumen before uptake by duodenal epithelial cells, or cellular ferritin donates its iron to duodenal epithelial ferritin relatively soon after uptake by these cells. It is also possible that both processes function concomitantly.

**Figure 4:**
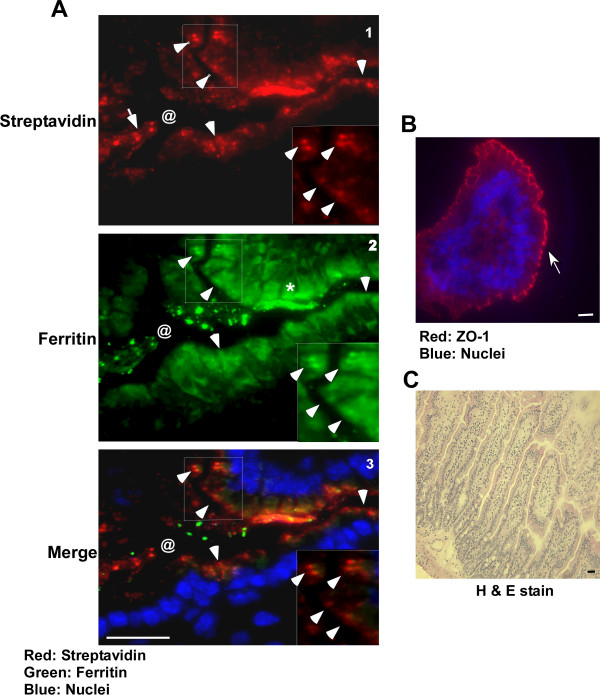
**Ferritin is endocytosed by duodenal enterocytes**. **(A) **Immunostaining of duodenal sections from mice fed biotin tagged, PK-treated, scrapie infected mouse brain homogenates with streptavidin-Texas-red shows uptake of biotin tagged material by intestinal epithelial cells (panel 1, arrow-heads). Unabsorbed reactive material is detected in the intestinal lumen (panel 1, arrow, @). Co-immunostaining with rabbit-anti-ferritin antibody followed by goat anti-rabbit-FITC shows co-localization of streptavidin and ferritin reactive material (panels 2 and 3, arrow-heads). Endogenous mouse ferritin cross-reacts with the antibody and is detected in most epithelial cells (panel 2, *). Bar: 10 μm. Inset shows enlarged image of the boxed area. **(B) **Immunostaining of adjacent section with antibody to ZO-1 demonstrates intact tight junctions in these samples (arrow). **(C) **Staining with H & E shows optimal preservation of tissue under these experimental conditions.

To distinguish between these possibilities, brain homogenate from scrapie infected mice was biotinylated with sulfo-NHS-biotin overnight at 4°C and treated with PK to enrich for ferritin and PrP^Sc^. Methanol precipitated proteins were resuspended in PBS and introduced to wild-type mice by gastric gavage. Control mice received the same volume of PBS. Following a chase of 2h, first 10 cm of the duodenum were harvested and processed for immunostaining. Reaction of sections with fluorescent streptavidin shows significant uptake of biotin tagged material by the duodenal epithelium (Figure 4A, panel 1, arrow-heads). Duodenal lumen (marked by @) shows similar streptavidin reactive material before internalization (panel 1, arrow). Immunoreaction with anti-ferritin antibody followed by FITC-tagged secondary antibody shows co-localization of ferritin and streptavidin within epithelial cells, indicating uptake of externally introduced ferritin (Figure 4A, panels 2 and 3, arrow-heads). Reaction for endogenous ferritin is also detected in epithelial cells (Figure 4A, panel 2, *). Immunoreaction of adjacent sections for ZO-1 shows intact tight junctions, ruling out non-specific uptake of ferritin by these cells (Figure 4B). Staining with hematoxylin and eosin demonstrates optimal preservation of duodenal epithelium under these experimental conditions (Figure 4C). Similar results were obtained with hamster brain ferritin.

### Iron depletion reduces efficiency of ferritin uptake by mouse intestinal epithelium

**Figure 5:**
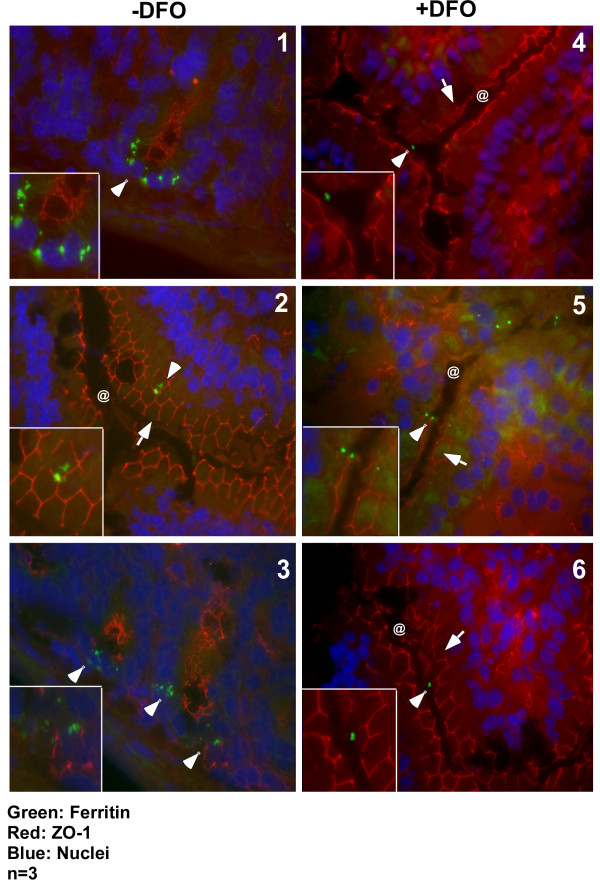
**Iron content of ferritin influences its uptake by duodenal enterocytes**. Immunostaining of 3 wild type mice fed -DFO and +DFO treated scrapie infected mouse brain homogenate with antibody to ZO-1 followed by anti-mouse-TRITC secondary antibody shows intact tight junctions (panels 2 and 4-6, arrow). Other sections do not show the epithelial cell layer and lack ZO-1 reactivity (panels 1 and 3). Reaction with anti-ferritin antibody followed by anti-rabbit-FITC shows more uptake of -DFO ferritin compared to the +DFO sample (compare panels 1-3 with 4-6, arrow-heads). Most ferritin from -DFO sample accumulates in intestinal crypts after 4 hours of chase (panels 1 and 3). Three representative sections are shown from a total of 6 different experiments performed by three different individuals. Bar: 10 μm. Insets show enlarged image of boxed areas.

The influence of iron content of ferritin on its uptake by mouse intestinal epithelial cells was checked by depleting iron from scrapie infected mouse brain homogenate as described above with DFO. Precipitated proteins from -DFO +DFO samples were re-suspended in PBS and introduced by gastric gavage to three sets of wild type mice starved overnight to deplete endogenous ferritin. Following a chase of 2 hours, first 10 cm of the duodenum were processed for immunostaining as above. Immunoreaction with antibody to ZO-1 followed by anti-mouse TRITC shows intact tight junctions along the luminal surface of duodenal epithelium (Figure 5, panels 2, 4, 5, and 6, arrows). Other sections show deeper areas highlighting intestinal crypts (Figure 5, panels 1 and 3). Reaction for ferritin followed by anti-rabbit-FITC shows significantly more uptake of ferritin from -DFO sample relative to the +DFO sample (Figure 5, panels 1-3 vs. 4-6, arrow-heads). Notable reactivity for ferritin is observed in the intestinal crypts of mice fed -DFO sample (Figure 5, panels 1 and 3, arrow-heads), indicating transport of ferritin from the epithelium to intestinal crypts. Intestinal crypts of mice fed +DFO sample did not show any reactivity (data not shown).

## Discussion

Our data indicate that brain ferritin from deer, elk, and sheep is transcytosed across a monolayer of human Caco-2 epithelial cells, representing an *in vitro *model of human intestinal epithelium. The efficiency of ferritin transport is modulated by its iron content, suggesting that ferritin serves as a biological source of iron. A similar selection for iron-rich ferritin is noted *in vivo *in mice administered iron depleted mouse brain homogenate. Since PrP^Sc ^and ferritin form a complex in diseased brain homogenates and resist degradation by digestive enzymes, these observations suggest that ferritin could serve as a mediator of PrP^Sc ^transport across intestinal epithelial cells regardless of homology between incoming PrP^Sc ^and host PrP^C^.

Previous attempts at understanding the mechanism of PrP^Sc ^transport across the intestinal epithelial barrier have uncovered several possible pathways such as migrating dendritic cells and intestinal M cells [36]. The involvement of laminin receptor (LRP/LR) dependent endocytosis of PrP^Sc ^has also been described [37,38]. LRP receptors have been identified on the intestinal brush border, and ingested PrP^Sc ^has been reported to co-localize with these receptors following ingestion. It is believed that majority of ingested PrP^Sc ^loses infectivity following digestion [39]. However, our observations suggest that majority of PrP^Sc ^and ferritin in the ingested material resist degradation by digestive enzymes, and are endocytosed by intestinal epithelial cells. Ferritin by itself is resistant to degradation and has been proposed as a source of dietary iron [30,31,40]. Whether association of PrP^Sc ^with ferritin increases its resistance to proteases in unclear at this time, but raises the possibility of co-transport with ferritin across the intestinal epithelium.

Regarding the uptake of ferritin, conflicting reports suggest either direct uptake followed by release of associated iron somewhere along the endocytic pathway, or release of iron in the intestinal lumen before uptake by epithelial cells through the DMT1 pathway. In support of the first hypothesis, receptors specific for ferritin uptake have been described on oligodendrocytes [41], activated B and T-cells, K562 cells, reticulocytes, and certain other cell lines [33], suggesting that ferritin is a common source of iron. Our observations indicate that ferritin is taken up by intestinal epithelial cells and accumulates in intestinal crypt cells before further transport or breakdown. Although our data fall short of identifying a specific receptor or demonstrating co-transport of PrP^Sc ^with ferritin due to technical reasons, our previous observations [7] and co-immunoprecipitation studies in this report leave little doubt that PrP^Sc ^and ferritin form a complex, and are likely to be co-transported across the intestinal epithelium.

This possibility raises significant public health concerns, especially due to the possibility of transmitting CWD prions from infected deer and elk to cattle and to the human food chain. Although PrP^Sc ^does not replicate unless the host PrP^C ^and incoming PrP^Sc ^share significant homology, transport with ferritin could establish a carrier state in non-homologous hosts, resulting in disease at a later time. On the other hand, the association of PrP^Sc ^with ferritin provides possible avenues to reduce exposure and infectivity by chelating available iron in the infected material. Such a treatment would have the combined effect of decreased ferritin uptake and increased degradation of PrP^Sc ^by digestive enzymes [this report, [34]]. Future research is necessary to understand the mechanism of PrP^Sc^-ferritin uptake by the intestine and practical ways to reduce infectivity due to accidental ingestion of PrP^Sc^.

## Competing interests

The authors declare that they have no competing interests.

## Authors' contributions

SRBS and XL carried out mouse experiments, AS conducted the radioactive iron feeding study and edited and revised the manuscript, and DD helped with certain sections of the manuscript. All authors read and approved the final manuscript.
